# Insights into the electronic and atomic structures of cerium oxide-based ultrathin films and nanostructures using high-brilliance light sources

**DOI:** 10.3762/bjnano.16.65

**Published:** 2025-06-10

**Authors:** Paola Luches, Federico Boscherini

**Affiliations:** 1 Istituto Nanoscienze – Consiglio Nazionale delle Ricerche, Via G. Campi 213/a, 41125 Modena, Italy; 2 Istituto Officina dei Materiali – Consiglio Nazionale delle Ricerche, Strada Statale 14, km 163.5, 34149 Trieste, Italy

**Keywords:** cerium oxide, free-electron lasers, thin films, X-ray absorption spectroscopy, X-ray photoelectron spectroscopy

## Abstract

High-brilliance light sources, such as synchrotrons and free-electron lasers, allow researchers to probe the structural, electronic, and dynamic properties of functional materials at an unprecedented level of detail. Techniques like X-ray photoelectron spectroscopy and X-ray absorption spectroscopy, can reveal atomic-scale information about material behavior under different conditions. This thorough understanding can be leveraged to optimize materials for various applications, including energy storage, catalysis, and electronics. This review focuses on cerium oxide, an important material for catalytic and energy applications, examining the application of high-brilliance light sources on model systems such as supported thin films and epitaxial nanostructures. We review selected studies exploiting the high energy resolution and sensitivity of synchrotron radiation-based X-ray photoelectron spectroscopy and X-ray absorption spectroscopy to explain the factors influencing the material’s reducibility, with particular focus on dimensionality effects and on metal–oxide interaction, and the interaction with molecules. The potential of studies conducted under ambient pressure conditions is highlighted, and, finally, the perspectives offered by the ultrahigh brilliance and ultrashort free-electron laser pulses for dynamic studies of the processes that take place upon photoexcitation are discussed.

## Introduction

Transition metal oxides in the form of thin films or nanostructures find extensive use in sustainable energy technologies [1–2]. They serve as active materials or supports for catalysts for various chemical reactions, essential to energy conversion, sensing, and environmental remediation [3–4]. Additionally, because of their often high efficiency at harnessing solar energy, they find application in photocatalysis and photovoltaics [5–6]. Optimizing these applications requires unravelling the often complex processes that influence functionality through an atomic-level description. To this end, materials are often studied as model systems, such as well-controlled supported thin films or nanostructures with simplified complexity compared to real systems, allowing the results from advanced experimental methods to be directly compared with theoretical simulations [7–8]. Thanks to their remarkable sensitivity, atomic selectivity, spatial and energy resolution, synchrotron radiation-based techniques, which utilize high-brilliance photon beams, have enabled refined characterization of the electronic, structural, and morphological properties of these materials, as well as the modifications they undergo under operating conditions. Furthermore, free-electron lasers (FELs), with orders-of-magnitude higher peak brilliance than synchrotrons, have made it possible to achieve temporal resolution of the order of a few tens of femtoseconds, facilitating an ultrafast, element-sensitive characterization of the dynamic processes occurring for example upon photoexcitation.

Among transition metal oxides, cerium oxide (or ceria) has unique redox properties, linked to the relative stability of Ce cations in the 4+ and 3+ oxidation states, which make the material highly effective in automotive catalysts and in the field of environmental remediation [9]. The related ability of the material to easily store and release oxygen also plays a key role in energy conversion technologies, including fuel cells and batteries [10–11]. Gas sensing applications of ceria-based materials are based on the modifications of the transport properties in the presence of specific gases, due to the redox reactions that take place on the surface [12–13]. An atomic level understanding of the structure–function relationship in this oxide is essential for guiding the design of efficient materials to optimize the performance of the applications. Studies on cerium oxide nanostructures and powders prepared by chemical synthesis methods are quite numerous, and they are typically carried out on systems with a marked and often unexplored complexity [14–17]. For studies on cerium oxide as model systems, such as low-index surfaces, thin films, and supported nanostructures, investigated using laboratory-based surface science methods, we refer the reader to existing reviews [18–20].

The aim of this work is to provide an overview of recent studies highlighting the advantages of using synchrotron and FEL radiation to achieve a refined understanding of cerium oxide-based materials, particularly when examined in the form of well-controlled thin films and nanostructures prepared by physical synthesis methods.

## Review

### Studies by X-ray photoelectron spectroscopy and related techniques

Synchrotron radiation-based X-ray photoelectron spectroscopy (XPS) has significantly advanced the characterization of low-dimensional cerium oxide structures by offering much higher sensitivity and energy resolution than conventional XPS [21–24]. In addition, the possibility to select photon energies in a broad range permits to tune the depth sensitivity of the method and to selectively probe the surface and or deeper layers, like buried interfaces.

Since the early studies of epitaxial cerium oxide films by Mullins and coworkers [22], it became clear that synchrotron radiation could provide high-resolution Ce 3d, Ce 4d, and valence band spectra. It is important to emphasize that the significantly higher brilliance of synchrotron radiation beams, compared with laboratory sources, provides much higher sensitivity towards diluted elements, such as low-concentration dopants and low-density metal NPs.

**Figure 1:**
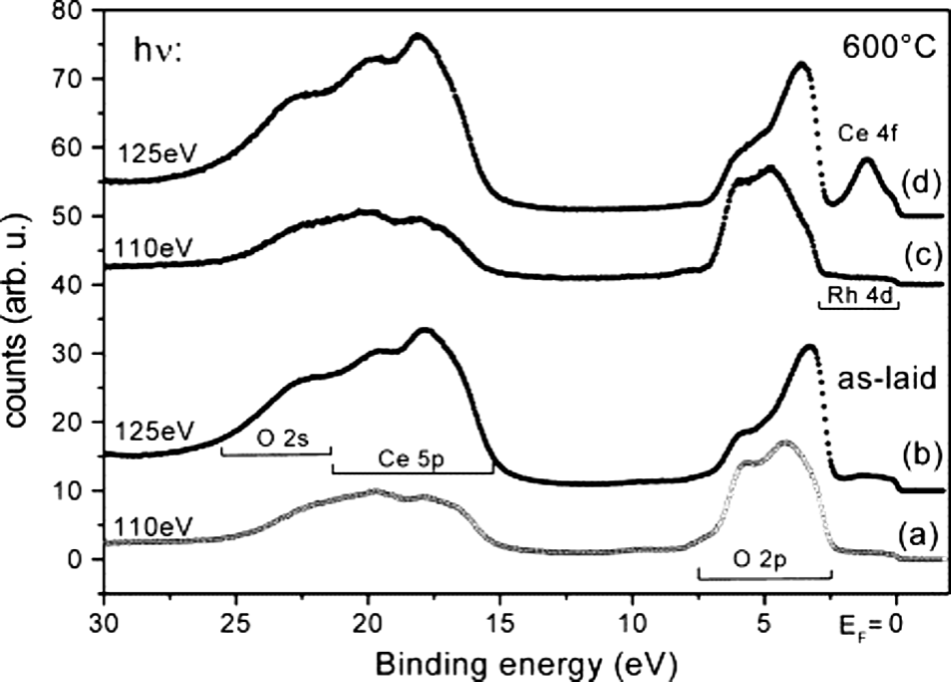
Valence band photoemission spectra of 1.3 MLE cerium oxide/Rh(111) as laid (a, b) and after annealing at 600 °C (c, d). Spectra (b) and (d) have been excited by a photon energy of 125 eV corresponding to the maximum of the Ce 4d→4f giant resonance (on-resonance), while spectra (a) and (c) have been excited with a photon energy of 110 eV (off-resonance). Reprinted from [23], Surface Science, vol. 520, by S. Eck; C. Castellarin-Cudia; S. Surnev; M. G. Ramsey; F. P. Netzer, “Growth and thermal properties of ultrathin cerium oxide layers on Rh(111)”, pages 173-185, Copyright (2002), with permission from Elsevier. This content is not subject to CC BY 4.0.

A significant step forward in the understanding of cerium oxide-based systems was introduced by the application of resonant photoemission to selectively probe valence band features related to Ce^4+^ and Ce^3+^ ions. This can be done by tuning the photon energy at specific resonances related to Ce 4d→Ce 4f^0^ (Ce^4+^) and Ce 4d→Ce 4f^1^ (Ce^3+^) electronic configurations at 110 and 125 eV, respectively [23,25–28]. Figure 1 reports valence band spectra from an ultrathin cerium oxide film before and after ultrahigh vacuum (UHV) annealing at 600 °C, acquired at the two resonant energies. Using a photon energy at the Ce^4+^-related resonance (110 eV), the spectrum shows only minor modifications after annealing, while if the Ce^3+^-related resonant energy is used (125 eV), a marked Ce 4f peak appears around 2 eV binding energy after the annealing. This demonstrates the significantly higher sensitivity to Ce^3+^ that can be achieved by exploiting resonant photoemission, as compared to non-resonant photoemission.

**Figure 2:**
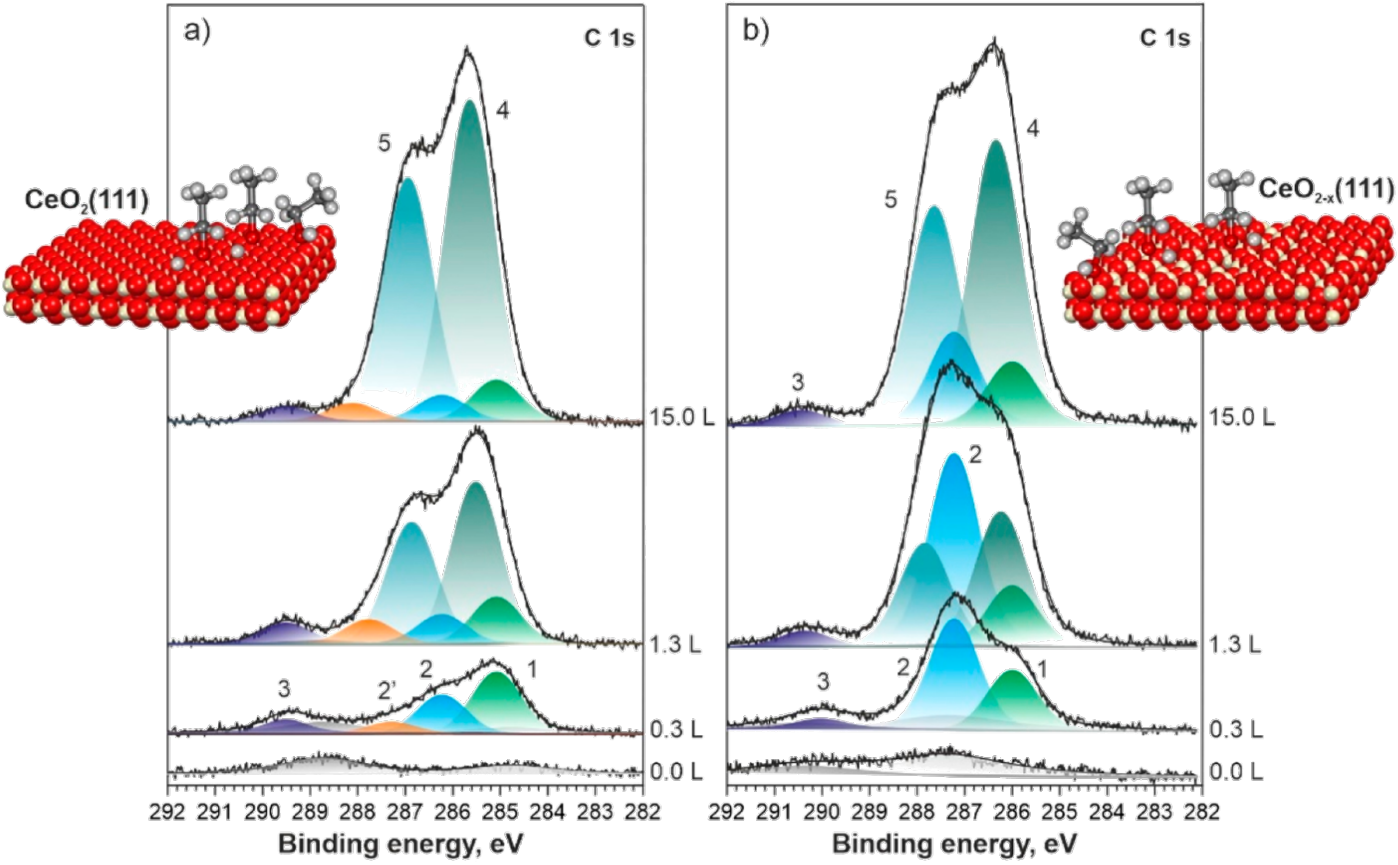
High-resolution synchrotron radiation C 1s XPS spectra on CeO_2_(111) (a) and CeO_2−_*_x_* (111) (b) for different ethanol exposures at 150 K. Reprinted from [35] (@ 2022 Y. Lykhach et al., published by IOP Publishing, distributed under the terms of the Creative Commons Attribution 4.0 International License, https://creativecommons.org/licenses/by/4.0).

Synchrotron radiation-based resonant photoemission has facilitated an accurate determination of the dependence of Ce^3+^ concentration on dimensionality [23]. The technique has also provided an accurate description of the charge transfer processes and hybridization occurring at the interface between cerium oxide and metals, either as substrates [23], as supported nanoparticles (NPs) [26–27] or as dopants [28]. The insight provided by such studies is highly relevant, since cerium oxide is often combined with metals in various applications. For example, it was possible to identify different types of interactions between Pt NPs and cerium oxide surfaces including electron transfer from Pt NPs to CeO_2_ and transport of oxygen atoms from ceria to Pt NPs, the latter occurring only when the ceria support surface is nanostructured [26]. In addition, thanks to its sensitivity, the method, when combined with other techniques, has provided quantitative information on the number of electrons transferred per particle to the support (Figure 2), enabling the optimization of the size of the supported active Pt catalyst [27]. This is a crucial factor in minimizing the concentration of critical and expensive noble metals in applications.

The possibility of tuning the kinetic energy of the photoelectrons by varying the photon energy has also enabled a much higher surface sensitivity than laboratory XPS by selecting a photoelectron kinetic energy close to the minimum of the inelastic mean free path. A study by Simon et al. on porous Pt-doped cerium oxide films prepared by direct liquid injection chemical vapor deposition, comparing conventional and synchrotron-radiation based XPS, has demonstrated that the Pt atoms are uniformly dispersed in the nanoparticles that form the film, while the Pt ions in the 2+ oxidation state are confined at the outermost layers [29].

The interplay of ceria surfaces with adsorbed molecules, a crucial factor in understanding reactivity, has become a significant research focus, also thanks to synchrotron-radiation-based XPS methods [30–32]. Initial studies were performed by exposing the surface of interest to the chosen molecule at UHV-compatible pressures. The interaction between cerium oxide and, for example, CO [21,33], SO_2_ [34], methanol [32], ethanol [35], and water [36] have been considered. The higher energy resolution and the tunable surface sensitivity of synchrotron radiation-based XPS, as compared to conventional XPS, permits a more accurate description of the different contributions to the spectra. Figure 2 shows high-resolution synchrotron-radiation-based C 1s spectra, for different ethanol exposures at 150 K, using a photon energy of 410 eV. At low exposure, the most intense peaks at 285.1 eV (1) and at 286.2 eV (2) on CeO_2_(111) and at 286.3 eV (1) and at 287.6 eV (2) on CeO_2−_*_x_*(111) are ascribed to methyl and alkoxy groups of adsorbed ethoxy species, respectively. The slightly different binding energies on the two surfaces are ascribed to the different Ce^3+^ concentrations in the two ceria films. The different intensity ratios between the methyl- and alkoxy-related peaks on the two surfaces are assigned to different adsorption and dissociation pathways for ethanol on a stoichiometric and a non-stoichiometric ceria surface. At higher exposures, the new peaks at 285.6 eV (4) and at 287.0 eV (5) on CeO_2_(111) and at 286.3 eV (4) and at 287.6 eV (5) on CeO_2−_*_x_*(111) are ascribed to physisorbed ethanol.

To identify the role of the interaction between ceria and metals, the adsorption and reaction of ethylene on Pt NPs supported on ceria were compared with the ones observed on a Pt(111) surface [31]. The oxide-supported NPs were shown to have an enhanced reactivity and additional reaction pathways [31]. Regarding the same system, the efficient decomposition of methanol and the resistance to poisoning have rationalized the observed high activity of the material in direct methanol fuel cells [37]. Regarding the Ni-CeO_2−_*_x_*(111) system, a study of the methanol reaction revealed that the strong metal–support interactions between Ni and CeO_2_ determines the high selectivity for CO_2_ production, instead of the formation of surface C or CO [38].

**Figure 3:**
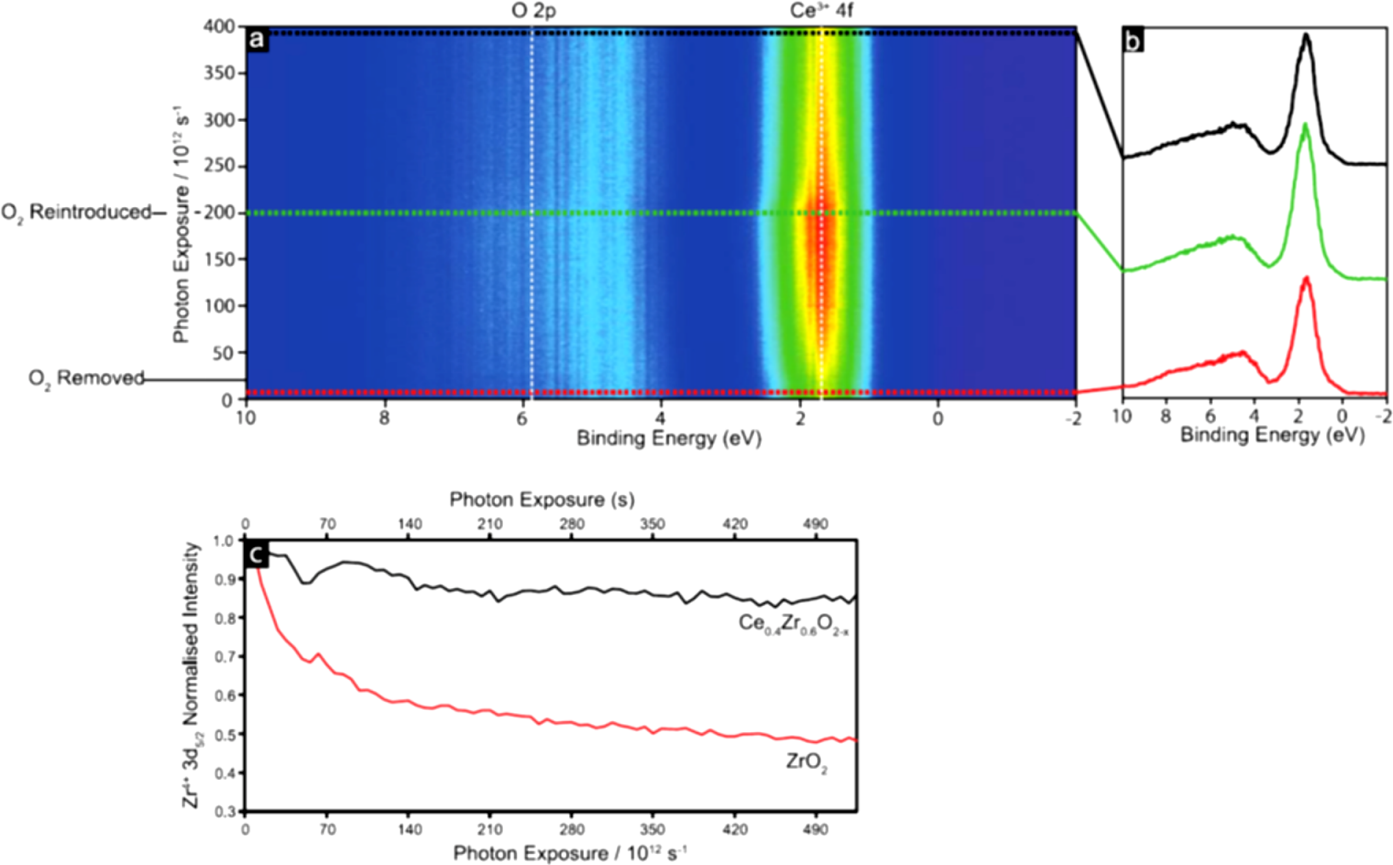
μ-XPS valence band spectra of a Ce_0.4_Zr_0.6_O_2−_*_x_* film acquired using a photon at the Ce^3+^-related Ce 4d→Ce 4f^1^ resonance (*h*ν = 120.8 eV), acquired at different photon exposure times after removal and subsequent reintroduction of oxygen (*P*_O2_ = 1 × 10^−7^ mbar) in the chamber. (b) Selected spectra of oxidized film (red), reduced film (green) and reoxidized film (black). (c) Evolution of the Zr^4+^ 3d_5/2_ intensity as a function of photon exposure time for a Ce_0.4_Zr_0.6_O_2−_*_x_* and a ZrO_2_ film. Adapted from [39], Surface Science, vol. 682, by M. Allan; D. Grinter; S. Dhaliwal; C. Muryn; T. Forrest; F. Maccherozzi; S.S. Dhesi; G. Thornton, “Redox behaviour of a ceria–zirconia inverse model catalyst”, pages 8-13, Copyright (2019), with permission from Elsevier. This content is not subject to CC BY 4.0.

Resonant photoemission detected by a spectroscopic photoemission and low-energy electron microscope (SPLEEM) was also used to acquire local information on the oxidation degree of cerium in mixed zirconia–ceria nanostructures supported on a Rh(111) single crystal [39]. Figure 3a shows a sequence of μ-XPS valence band spectra acquired using a photon at the Ce^3+^-related Ce 4d→Ce 4f^1^ resonance (*h*ν = 120.8 eV) on a Ce_0.4_Zr_0.6_O_2−_*_x_* film upon removal and reintroduction of oxygen (*P*_O2_ = 1 × 10^−7^ mbar) in the experimental chamber. When oxygen is removed (red spectrum in Figure 3b), the intensity of the Ce^3+^-related feature at ≈2 eV decreases with photon exposure, while when oxygen is reintroduced (red spectrum Figure 3b), its intensity re-increases. The comparison of the time evolution of the Zr^4+^-related 3d_5/2_ μ-XPS intensity acquired on a Ce_0.4_Zr_0.6_O_2−_*_x_* and on a ZrO_2_ film is reported in Figure 3c. The exposure to the photon beam induces a much more pronounced decrease of Zr^4+^ intensity in the ceria–zirconia mixed oxide film than in pure zirconia. This was ascribed to a synergy between the two oxides inducing an oxygen transfer from ceria to zirconia upon reduction using soft X-ray irradiation. The observed effect was identified as responsible for the enhanced catalytic activity of mixed ceria–zirconia materials in the applications [39].

Photoemission is a technique with a sensitivity limited to the topmost surface layers. In typical resonant photoemission experiments on ceria, performed with photon energies close to the Ce N_45_ adsorption edge, the probed valence band photoelectrons have a kinetic energy close to the minimum of their inelastic mean free path, and the information comes from the topmost one or two atomic layers. If photons in the hard X-ray range are used in hard X-ray photoelectron spectroscopy (HAXPES) and photoelectrons with kinetic energies of a few kiloelectronvolts are probed, the depth sensitivity can be extended to the topmost 5–10 nm of the sample. The possibility to select a much higher photon energy than at typical laboratory sources has also permitted to acquire and analyze Ce 2p_3/2_ core level spectra using HAXPES at photon energies higher than the core-level binding energy of 5723 eV [24]. The analysis of Ce 2p core level spectra enables an accurate determination of Ce^3+^ and Ce^4+^ concentration due to the absence of spectral overlap between spin–orbit split lines [24]. In contrast, Ce 3d spectra exhibit five partially overlapped spin–orbit split components originating from different final states, which complicate the analysis [40].

Synchrotron radiation-based photoemission has proven to be significantly more versatile than XPS/UPS with laboratory sources. However, the information it provides primarily pertains to the surface atomic layers of the sample, as it relies on detecting electrons that strongly interact with materials.

### Studies by X-ray absorption spectroscopy and related techniques

X-ray absorption spectroscopy (XAS) has provided complementary information to that obtained by synchrotron radiation-based photoemission on cerium oxide-based materials. The depth sensitivity of XAS can be tuned on a much wider range than XPS by choosing the desired absorption edge and by selecting a specific detection mode. Moreover, complementary information on the electronic properties and on the local atomic structure can be obtained from the analysis of XAS data. X-ray absorption near-edge spectroscopy (XANES), analyzing the signal within the first few tens of electronvolts above the absorption edge, provides information mainly on the density of empty states of the investigated sample. In contrast, the extended energy range X-ray absorption fine structure (EXAFS), up to a few hundred electronvolts above the absorption edge, is sensitive to the local atomic structure around the absorbers.

**Figure 4:**
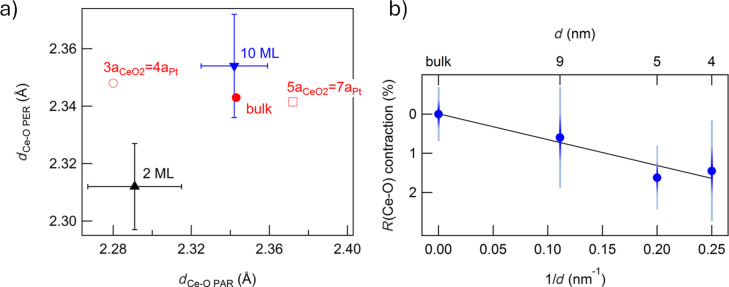
(a) Ce–O interatomic distances for 2 ML and 10 ML CeO_2_ films evaluated by fitting Ce L_3_-edge EXAFS with the electric field parallel (*d*_Ce−O PAR_) and perpendicular (*d*_Ce−O PER_) to the sample surface. The value expected for a bulk (red dot) and assuming in-plane 3a_CeO2_ = 4a_Pt_ and 5a_CeO2_ = 7a_Pt_ coincidence are also shown. Reprinted with permission from [41], Copyright 2013 American Chemical Society. This content is not subject to CC BY 4.0. (b) Contraction of the Ce–O interatomic distance in CeO_2_ NPs of different diameter as function of the reciprocal average diameter evaluated by fitting Ce L_3_-edge EXAFS. The solid line is a linear fit of the data. Used with permission from [42] (“Contraction, cation oxidation state and size effects in cerium oxide nanoparticles”, by J. S. P. Cresi et al., Nanotechnology, vol. 28, issue 49, article no. 495702, published on 13 November 2017; https://iopscience.iop.org/article/10.1088/1361-6528/aa926f; © 2017 IOP Publishing Ltd; permission conveyed through Copyright Clearance Center, Inc. All rights reserved. This content is not subject to CC BY 4.0.

A polarization-dependent Ce L_3_-edge EXAFS study of ultrathin epitaxial cerium oxide films on Pt(111) demonstrated the influence of the substrate in 2 monolayer (ML) films. Figure 4a shows the Ce–O in-plane and out-of-plane distances obtained from the analysis of EXAFS data acquired on a 2 ML and on a 10 ML ceria film. Figure 4a also reports the values expected for bulk ceria and for epitaxially distorted ceria, assuming that the film adopts an in-plane 3a_CeO2_ = 4a_Pt_ or a 5a_CeO2_ = 7a_Pt_ coincidence to compensate from the large lattice mismatch with the Pt support. The values of the expected out-of-plane Ce–O distances in the two cases are calculated assuming the bulk elastic constants. The 2 ML film adopts an epitaxial in-plane compression to match the substrate in a dominant 3a_CeO2_ = 4a_Pt_ (Figure 4a). The out-of-plane Ce–O bonds in the ultrathin film appear shorter than expected considering the bulk elastic constants, possibly because of the reduced dimensionality of the system. In contrast, the epitaxial compression is completely relaxed in the 10 ML film, which assumes interatomic distances compatible with the bulk value (Figure 4a) [41].

In analogy, for CeO_2_ NPs of different diameters prepared using physical synthesis the analysis of Ce L_3_-edge EXAFS data demonstrated a progressive contraction of the Ce–O distance with respect to the bulk value with decreasing diameter (Figure 4b). The contraction showed a linear dependence on the surface-to-volume ratio (Figure 4b), and it was ascribed to a compressive strain arising from reduced dimensionality [42]. This effect was theoretically predicted [43], but it was never experimentally observed on chemically synthesized NPs, where an expansion typically occurs [44–46], possibly due to the higher Ce^3+^ surface concentration compared to physically synthesized NPs.

The short- and long-range structural modifications associated with thermal reduction in CeO_2_/Pt(111) films, as well as the influence of the Pt substrate’s proximity, were investigated using XANES/EXAFS combined with surface X-ray diffraction (SXRD) [47]. A strong interaction between cerium oxide and platinum was identified and associated to the formation of a Ce–Pt alloyed interfacial phase exhibiting a (2 × 2) periodicity [47]. The influence of the substrate on the stability and reactivity of supported ceria nanoislands has also been investigated by Ce M_5_ XANES in the case of Au(111) [48]. A loss of redox activity accompanied by an irreversible amorphization was observed at high reduction temperatures, while a partial decomposition of the ceria nanoislands to metallic cerium was found to occur under milder conditions than on Pt(111) or other metal substrates [48].

**Figure 5:**
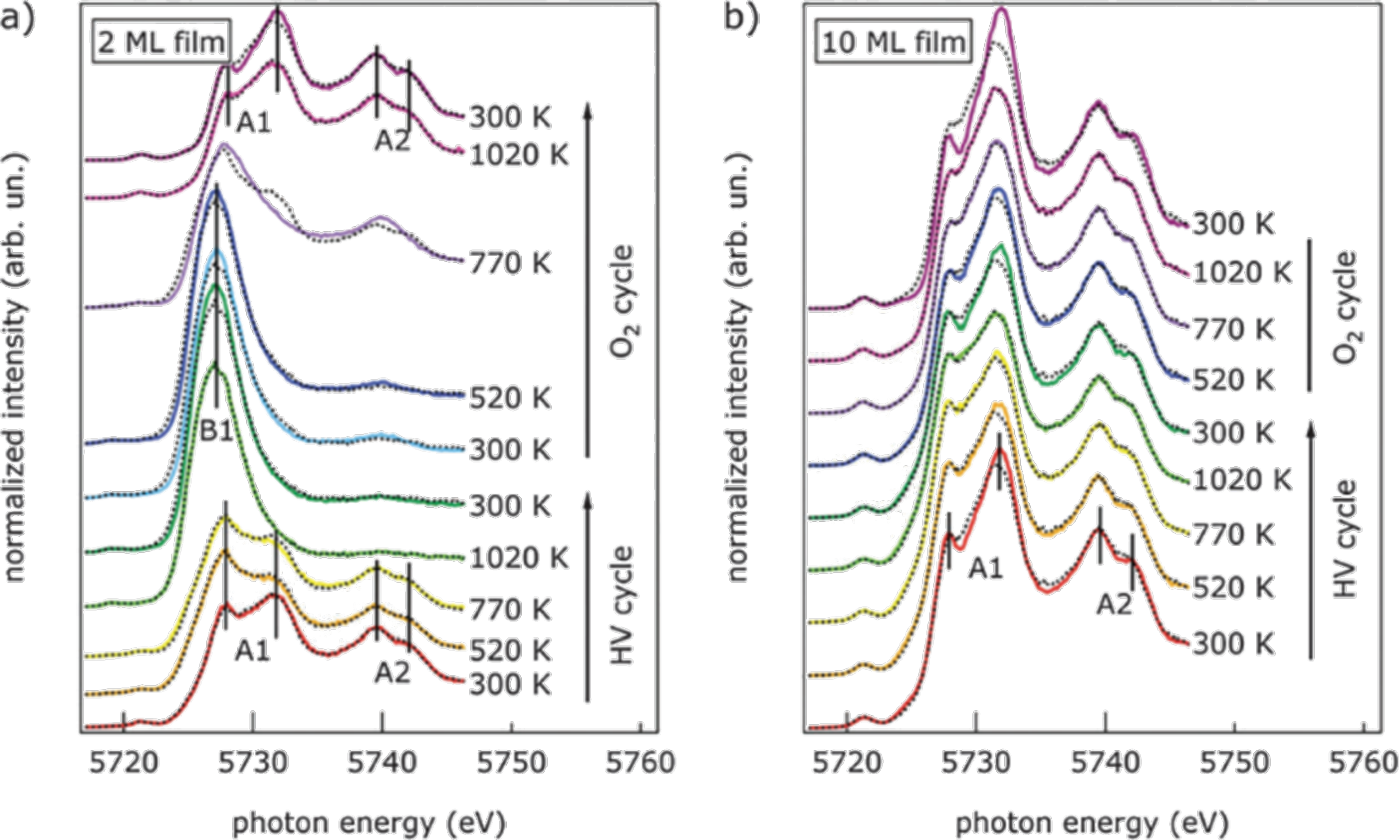
Ce L_3_-edge HERFD-XANES spectra of (a) 2 ML and b) 10 ML CeO_2_ films acquired during the thermal cycles in high vacuum and in oxygen-rich atmosphere. The characteristic Ce^4+^-(A1 and A2) and Ce^3+^-(B1) related peaks are marked. The fits with linear combinations of the Ce^3+^ and Ce^4+^ references are shown as dashed lines. Reprinted from [49] with permission of the Royal Society of Chemistry (“Electronic properties of epitaxial cerium oxide films during controlled reduction and oxidation studied by resonant inelastic X-ray scattering” by G. Gasperi et al., Phys. Chem. Chem. Phys. vol. 18, issue 30, article no. 20511, **©** 2016); permission conveyed through Copyright Clearance Center, Inc. This content is not subject to CC BY 4.0.

High-energy resolution fluorescence detected (HERFD)-XANES was used to achieve a more detailed understanding of the processes accompanying thermal reduction in ultrathin Pt(111) supported cerium oxide nanostructures [49]. In this technique, the incident photon energy is scanned across an absorption edge, while a spectrometer selects the energy of the emitted photons [50]. If a specific fluorescence decay with a sufficiently narrow energy bandwidth is selected, an energy resolution higher than that limited by core–hole lifetime broadening can be achieved. For Ce L_3_-edge absorption (5715–5750 eV), the emission spectrometer was tuned to the L_α1_ channel at 4840 eV to obtain HERFD-XANES, which showed to be sensitive to the electronic configurations of the Ce 4f levels and 5d band [51–52]. This approach, applied to Pt-supported cerium oxide films of 2 and 10 ML thickness, permitted to clearly resolve the fine structure of the two groups of white lines in Figure 2, ascribed to screened (A1) and unscreened (A2) 2p–5d transitions with the additional splitting due to the fine structure of the 5d band due to crystal field effects. As shown in Figure 2a, the intensity and shape of the A1 feature of the 2 ML sample are significantly modified by thermal treatment in vacuum up to 770 K. At 1020 K, a new peak, labeled B1 in Figure 5a, characteristic of Ce^3+^, appears. The A1 and A2 structures reappear after heating in O_2_, and recover a shape close to the initial one after sample cooling to RT in O_2_. The spectra of the 10 ML film (Figure 5b) instead show only minimal changes with the same thermal treatments in vacuum [49]. The study allowed the authors to conclude that in 2 ML films vertical confinement and/or charge transfer from the platinum substrate, determine a higher reducibility than in 10 ML films [49]. This is evidenced by the greater Ce^3+^ concentration formed during thermal treatments in high vacuum up to 770 K and by the full reversibility of the process upon thermal treatments in O_2_ (Figure 5) [49].

The interaction between cerium oxide and Ag NPs of varying sizes supported on a cerium oxide film were investigated using EXAFS at the Ag K-edge [53]. The NPs exhibited an Ag–Ag interatomic distance contracted by 3–4% compared to the bulk value [53]. The contraction was mainly ascribed to dimensionality effects, with epitaxial effects having a minor role. Additionally, the Ag–O interatomic distance at the interface between the NPs and the supporting oxide was found to decrease with decreasing NP size [53], possibly due to a stronger interaction between Ag and cerium oxide in smaller NPs, resulting from a greater charge transfer per atom [54].

A further possibility offered by synchrotrons is to measure spatially resolved Ce M-edge absorption spectra in photoemission electron microscopy (PEEM) mode, in which the photon energy is scanned across the XAS edge and the intensity of the secondary electrons is detected using a PEEM. This allowed to image the shape and size of ceria nanoislands on Ru(0001) and to probe and compare the oxidation state in selected areas with sub-micrometer spatial resolution [55]. In addition, the morphology of zirconia–ceria mixed oxides supported on Rh(111) and the oxidation states of the two oxides, individually and in the mixed phase, were determined [39].

### Studies at ambient pressure

The identification of active sites in catalysts is a crucial problem in view of the optimization of catalyst efficiency and selectivity. The possibility of carrying out spectroscopic studies under conditions as close as possible to ambient pressure has largely contributed to this goal. The application of these methods to model systems allowed for a comparison with simulations that were extremely valuable for a reliable interpretation of the results.

Ambient pressure XPS studies can be performed using a differentially pumped analyzer, which collects electrons at ambient pressure while the body of the analyzer remains under UHV conditions. Such instruments, installed at synchrotron radiation facilities and applied to ceria-based model systems have given an important contribution to understanding the surface chemistry of specific reactions. Coexisting Pt and ceria nanoparticles supported on TiO_2_(110) were found to exhibit a unique reactivity for the binding and dissociation of CO_2_, not observed on the TiO_2_(110) surface, on ceria supported on TiO_2_(110), or on bulk platinum surfaces [56]. In the same system, the high ability to bind and activate CO_2_ was exploited for the hydrogenation of CO_2_ to methanol, with the addition of water facilitating the production of ethanol [57].

**Figure 6:**
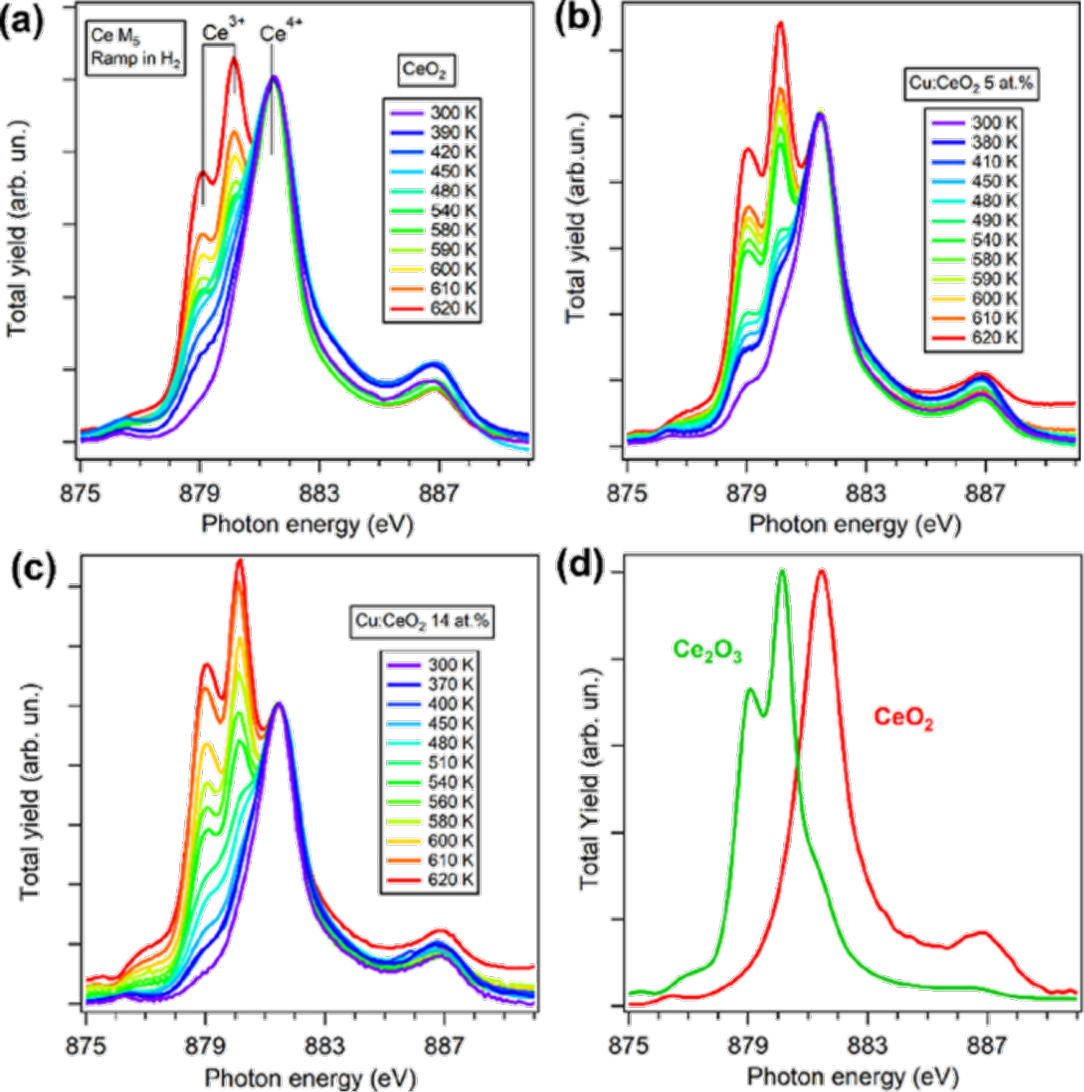
Ce M_5_-edge XANES spectra of (a) an undoped CeO_2_ film, (b) a Cu:CeO_2_ 5 atom % doped film and (c) a Cu:CeO_2_ 14 atom % doped film acquired at different *T* in H_2_ at ambient pressure. (d) Reference spectra measured for CeO_2_ and Ce_2_O_3_. Reprinted with permission from [58], Copyright 2024 American Chemical Society. This content is not subject to CC BY 4.0.

Given the high penetration depth of hard X-rays in materials, XAS in the hard X-ray range is much easier than XPS to be carried out under ambient pressure conditions; it provided valuable insight into the working principles of ceria-based catalysts. In the soft X-ray range, the relatively high absorption of X-rays by gases or liquids at ambient pressure and the limited inelastic mean free path of secondary electrons, when total electron yield is used as a detection mode, put some constraints on the application of ambient pressure XAS in the soft X-ray range. Reaction cells with an ultrathin membrane that confines the gas in a narrow region extremely close to the sample surface were applied to the study of Cu- and Fe-doped cerium oxide films during thermal treatments in hydrogen at ambient pressure [58]. The combination of ambient pressure XANES and gas chromatography was employed to correlate in real time the changes of the chemical state of Cu, Fe, and Ce cations with oxygen vacancy and water formation during thermal treatments in hydrogen at ambient pressure [58]. The pure ceria film, in the Ce^4+^ oxidation state with a dominant Ce L_3_-edge XANES feature at 881 eV, showed a progressively increasing relative intensity of the Ce^3+^-related features at 878.8 and 879.9 eV with increasing temperature in H_2_ pressure (Figure 6a compared with Figure 6d). In Cu-doped films, as the Cu concentration increases, the same treatment leads to a progressively higher intensity of the Ce^3+^-related features (Figure 6b,c). This evidence, combined with the evolution of the Cu oxidation state and with gas chromatography, suggested that at moderate temperatures, H_2_ dissociation is favored by the presence of Cu^1+^ sites, and at higher temperatures, water is desorbed from the surface with the uptake of oxygen from cerium oxide [58].

The same method was used to investigate the same system also during exposure to water and to laser light at different temperatures [59]. Also in this case the Cu dopant ions were found to be active in modifying the electronic structure of CeO_2_ and in enabling a more efficient hydrogen production at lower temperatures, as compared to the pure oxide [59].

### Dynamic studies of photoexcited states

The advent of X-ray free electron lasers has opened entirely new research pathways in the field of oxides for energy applications. The ultrashort and ultraintense photon beams with variable energy across a broad range can provide an accurate description of the processes that accompany any perturbation, for example, photoexcitation, in materials. The element sensitivity and the time resolution of the order of tens of femtoseconds and below can be exploited to obtain insight into the processes following photoexcitation in photocatalysts, important for the rational optimization of these materials’ efficiency.

In ceria, as well as in other semiconducting oxides, the formation of photoinduced small polarons after bandgap photoexcitation, was hypothesized based on optical pump–probe spectroscopy experiments [60]. These quasi-particles, which originate from the coupling between photoexcited charge and lattice distortions induced by the extra charges themselves, largely influence the transport properties of the material.

A study by Katoch et al. investigated the dynamics of photoexcited electron and hole polarons in a cerium oxide single crystal and in a nanocrystal using FEL-based pump–probe XANES at the Ce M_5_ and O K edges detected in total electron yield mode [61]. The samples contained a non-negligible concentration of Ce^3+^ sites within the probed depth. The authors found evidence for electron polaron formation within 0.7 ps, followed by fast decay within a few picoseconds and remnant effects persisting for longer than 1000 ps. In nanocrystals, the holes were found to have longer lifetimes than in single crystals, and they appeared mainly located close to the surface [61]. A different study performed on clean and stoichiometric cerium oxide films by pump–probe XAS at the Ce L_3_ edge in the XANES and EXAFS range, detected in the total fluorescence yield mode, provided proof for photoinduced polaron formation by clearly demonstrating a time correlation between the electronic and structural modifications within 500 fs and by finding a structural distortion quantitatively compatible with the one foreseen for a photoinduced polaron [62]. The photoinduced polarons were found to have a lifetime that exceeded 300 ps. The two studies on samples with different defectivity and using techniques with different probing depths suggest that defects can act as polaron trapping and recombination sites and that the excited charge dynamics can be different on the surface and in the bulk of the investigated oxide.

**Figure 7:**
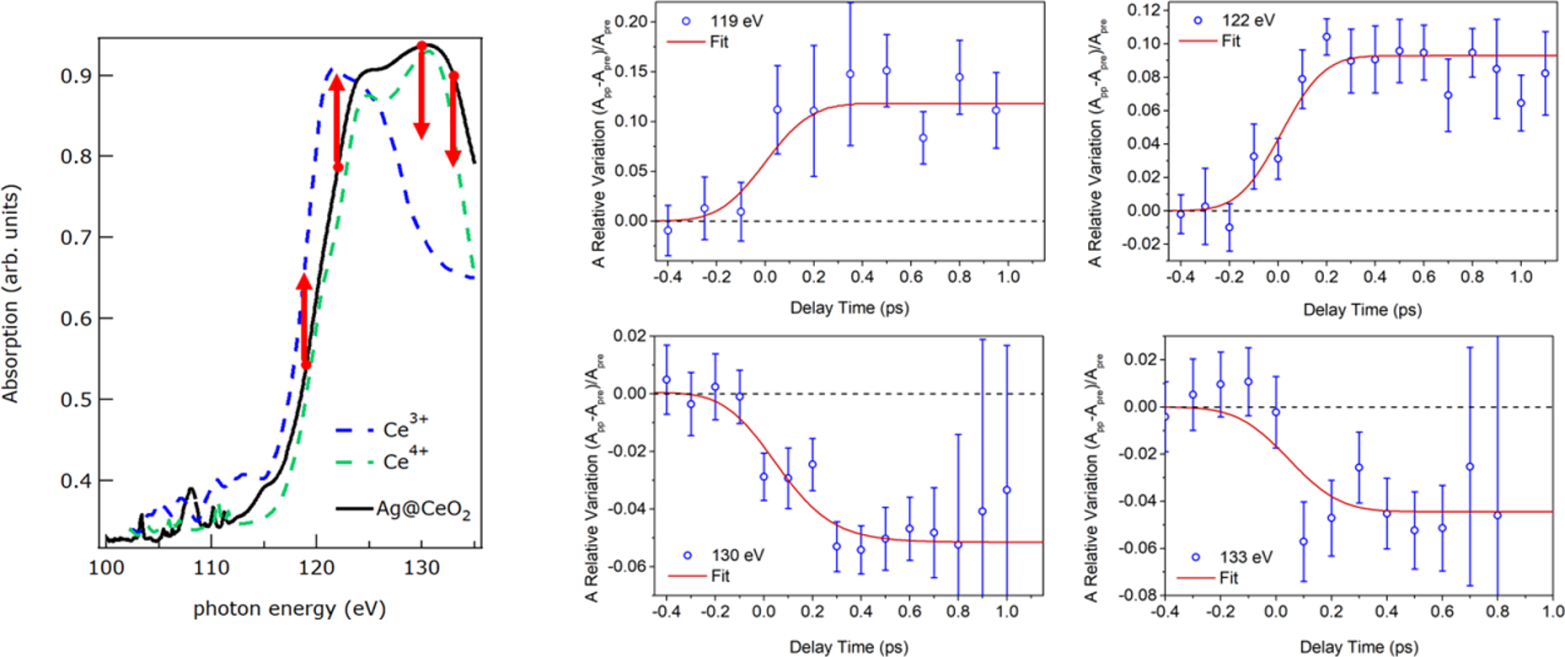
Left: Ce N_4,5_ XAS absorption spectra measured in transmission mode for the CeO_2_ film with embedded Ag NPs (solid black line). The spectra of Ce^4+^ (dashed green line) and Ce^3+^ (dashed blue line) reference samples, taken from the literature [64], are also reported. The red arrows indicate the selected FEL energies used to probe the variations of absorption with pump–probe delay time reported in the four right panels, and the direction of the observed variations. Reproduced from [63] (© 2021 J.S. Pelli Cresi et al., published by American Chemical Society, distributed under the terms of the Creative Commons Attribution 4.0 International License, https://creativecommons.org/licenses/by/4.0).

The chemical sensitivity of FEL was also exploited to investigate the electron transfer process in a composite plasmonic/semiconductor system, based on silver NPs embedded in a CeO_2_ film [63]. Pump–probe XANES measurements were performed at the Ce N_4,5_ edge upon photoexcitation of plasmonic resonances in the metal NPs by visible laser pulses at an energy lower than the cerium oxide bandgap. The four panels on the right of Figure 7 show the variation of the transient absorption as a function of the pump–probe delay time at selected FEL photon energies across the Ce N_4,5_ edge. The left panel of Figure 7 shows the steady-state Ce N_4,5_ XANES acquired using synchrotron radiation on the composite film and two reference spectra of Ce^4+^ and Ce^3+^ from literature [64]. Also, it evidences the photon energies used for the pump–probe FEL measurements. The transient XANES intensity after pumping the Ag plasmonic resonance, exhibits an increase by about 10% at 119 and 122 eV and a decrease by about 5% at 130 and 133 eV. The changes in the transient XANES intensity occur within the first few hundred femtoseconds and persist up to 1 ps delay time between the pump and the probe. The observed ultrafast changes across the Ce N_4,5_ absorption edge are compatible with a transient reduction of Ce ions in the film, as shown by the left panel of Figure 7, and they demonstrate that the resonantly excited plasmonic Ag NPs transfer electrons to the Ce atoms of the CeO_2_ film through a highly efficient electron-based mechanism [63]. The importance of this finding is that it provided a direct explanation for the observed sensitization of wide-bandgap oxides, such as cerium oxide, to the visible range through the coupling with suitable plasmonic metal NPs. The NPs convert the resonantly absorbed visible photons into excited charges in the oxide, which have similar properties as the ones induced by direct UV photoexcitation across the bandgap.

## Conclusions and Perspectives

The present review reports the advances brought by the use of high-brilliance photon beams generated by synchrotron radiation and FEL sources in the study of cerium oxide-based materials in the form of model systems. Synchrotron radiation-based spectroscopy methods have provided a higher energy resolution and a higher sensitivity to elements with low concentration than laboratory sources, allowing for the study of the properties of ultrathin films, of the effect of low-concentration dopants, and of the interaction with supported NPs. A quantitatively accurate description of the material in its static form has thus been achieved. The possibility to apply ultrashort and ultraintense FEL pulses has opened exciting perspectives for element-sensitive and time-resolved studies of the photoinduced processes in cerium oxide-based and related systems. The first results obtained by pump–probe XAS are very encouraging in view of a more extensive application of other ultrafast FEL-methods, for example, pump–probe photoemission, pump–probe resonant X-ray emission spectroscopy, or pump–probe X-ray diffraction, to more complex systems like highly doped and ternary oxides, or to systems with variable and well-controlled defect densities and architecture, also under operando conditions. Optimizing the lifetime of photoexcited charges, but also understanding the mechanisms and the extent of their spatial propagation, are extremely relevant questions in order to design materials with optimal efficiency.

## Data Availability

Data sharing is not applicable as no new data was generated or analyzed in this study.
